# Bone Marrow Stem/Progenitor Cells Attenuate the Inflammatory Milieu Following Substitution Urethroplasty

**DOI:** 10.1038/srep35638

**Published:** 2016-10-20

**Authors:** Joceline S. Liu, Matthew I. Bury, Natalie J. Fuller, Renea M. Sturm, Nida Ahmad, Arun K. Sharma

**Affiliations:** 1 Department of Urology, Northwestern University Feinberg School of Medicine, Chicago, IL USA; 2 Division of Pediatric Urology, Ann & Robert H. Lurie Children’s Hospital of Chicago, Chicago, IL USA; 3 Simpson Querrey Institute for BioNanotechnology, Northwestern University, Chicago, IL USA; 4 Department of Biomedical Engineering, Northwestern University, Chicago, IL USA; 5 Stanley Manne Children’s Research Institute, Chicago, IL USA

## Abstract

Substitution urethroplasty for the treatment of male stricture disease is often accompanied by subsequent tissue fibrosis and secondary stricture formation. Patients with pre-existing morbidities are often at increased risk of urethral stricture recurrence brought upon in-part by delayed vascularization accompanied by overactive inflammatory responses following surgery. Within the context of this study, we demonstrate the functional utility of a cell/scaffold composite graft comprised of human bone marrow-derived mesenchymal stem cells (MSC) combined with CD34+ hematopoietic stem/progenitor cells (HSPC) to modulate inflammation and wound healing in a rodent model of substitution urethroplasty. Composite grafts demonstrated potent anti-inflammatory effects with regards to tissue macrophage and neutrophil density following urethral tissue analyses. This was accompanied by a significant reduction in pro-inflammatory cytokines TNFα and IL-1β and further resulted in an earlier transition to tissue remodeling and maturation with a shift in collagen type III to I. Grafted animals demonstrated a progressive maturation and increase in vessel size compared to control animals. Overall, MSC/CD34+ HSPC composite grafts reduce inflammation, enhance an earlier transition to wound remodeling and maturation concurrently increasing neovascularization in the periurethral tissue. We demonstrate the feasibility and efficacy of a stem cell-seeded synthetic graft in a rodent substitution urethroplasty model.

Male urethral stricture disease is prevalent with an incidence estimated between 200 and 1200 cases per 100,000 individuals and has a substantial impact on quality of life and healthcare costs^1^. Urethroplasty is considered the gold-standard treatment in the definitive management of urethral stricture disease with the incorporation of graft in substitution urethroplasty in cases of longer strictures^2^. Although buccal mucosal graft has emerged as the most commonly employed graft with long-term success rates ranging from 80–90%, pain and morbidity with harvest and donor-site availability in cases of extensive or recurrent strictures remains an obstacle^2^,^3^. Patients with underlying local tissue compromise, including previous urethral surgery, pelvic radiation, pelvic trauma, lichen sclerosus and hypospadias, pose an especially challenging group to manage with a higher risk of recurrent stricture formation secondary to poor vascularization and chronic inflammation^4^,^5^,^6^. Impaired wound healing, excessive scar formation and persistent inflammatory responses with urethral reconstruction remain a challenge for urologists.

With advances in the field of tissue engineering, multiple strategies have been proposed for urethral regeneration and reconstruction. These include the use of synthetic and biologic scaffolds in the presence or absence of exogenous cell seeding. These models have been utilized with variable success rates in several animal models but have been hampered by inherent variability in composition, compliance and structural reliability of available scaffolds. This has been coupled with the inability to modulate inflammation in response to graft material while concurrently promoting tissue regeneration^7^,^8^. Suitable synthetic scaffolds, which are reproducible, biodegradable and capable of mimicking mechanical characteristics of the native urethra, are an attractive option for alternative graft material in urethroplasty. In the rat bladder augmentation model, the use of a synthetic scaffold material seeded with human bone marrow derived mesenchymal stem cells (MSC) and CD34+ hematopoietic stem/progenitor cells (HSPCs) has contributed to two facets of urothelial tissue engineering that have been lacking to date. This model has demonstrated striking neovascularization and modulation of inflammatory response in healing bladder tissue that are attractive in other models as well^9^,^10^. We therefore explore the application of the elastomeric synthetic scaffold [poly(1,8-octanediol-co-citric acid), (POC)], seeded with MSC/CD34+ HSPC as a cell/scaffold composite graft in the substitution urethroplasty rat model. Hence we investigate the effects of this composite graft on urethral wound healing, inflammatory response, neovascularization, and fibrosis. Applying epitope-defined stem cell populations to graft material that have proven effective in the bladder augmentation model for promotion of angiogenesis, decreased collagen accumulation and attenuation of inflammatory response to the urethra may identify ways to alter the healing process in the setting of substitution urethroplasty thus decreasing risk of re-stricture and scarring.

We hypothesize that by using this scaffold seeded with specific stem cell populations, we may be able to enhance the healing and reconstitution of the urethra postoperatively when the graft is incorporated. An associated decreased risk of stricture and scarring would allow us to improve the rates of long-term success of urethroplasty and reduce the need for repeated surgeries.

## Materials and Methods

### Substitution urethroplasty rat model

**Figure 1:**
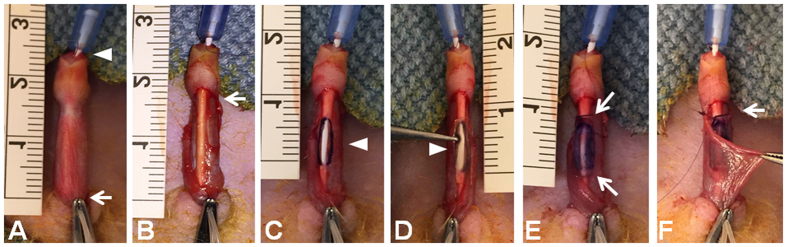
Description of substitution urethroplasty in a rat model. (**A**) A 22G venipuncture catheter was advanced into the urethra (arrowhead). A tourniquet at the base of the penis was applied (arrow). (**B**) A circumcising incision is made (arrow), degloving the penis. (**C**) A 6 mm ventral urethral incision was made (arrowhead). (**D**) POC graft (2 mm × 8 mm × 0.15 mm, highlighted with arrowhead), either unseeded or seeded with MSC/CD34+ HSPC, (**E**) was sutured to the cut urethral mucosal edges circumferentially with running 7-0 PDS (apices indicated by arrows). (**F**) The circumcision incision was closed with 7-0 PDS (arrow), catheter was removed, tourniquet removed and Bacitracin applied.

The elastomeric scaffold POC, was synthesized and cell-seeded as previously described^10^,^11^. Briefly, polymerized POC was cut into 2 mm × 8 mm × 0.15 mm (width × length × thickness) scaffolds and then seeded with 15,000 bone marrow derived MSC/cm^2^ followed by 25,000 bone marrow derived CD34+ HSPC/graft (Lonza Inc. MD, USA). If cell-seeded scaffolds demonstrated less than ideal cell growth as determined by light microscopy, re-seeding of either MSCs or CD34+ HSPCs was performed as needed to achieve cellular confluence prior to urethroplasty. Nude athymic male rats (12 weeks old, Charles River Laboratories; MA, USA) were anesthetized with inhaled isoflurane (2%) and underwent urethroplasty procedure. Cotransplantation of MSCs and CD34+ HSPCs populations was performed based on previously published data establishing clear synergistic effects^10^,^11^. In our experience, CD34+ HSPC seeding in isolation demonstrates poor cell survival on grafts. Surgical procedure is detailed in Fig. 1. A 22G venipuncture catheter was advanced into the urethra and secured to the glans using a 7-0 polydioxanone suture (PDS). A tourniquet at the base of the penis was applied and a circumcising incision is made, degloving the penis. A 6 mm ventral urethral incision was made. POC graft, either unseeded or seeded with MSC/CD34+ HSPC, was sutured to the cut urethral mucosal edges with running 7-0 PDS. Venipuncture catheter was retracted to the glans, with retrograde injection of saline to check for leaks. If necessary, additional simple interrupted sutures were applied to close the leak. The circumcision incision was closed with 7-0 PDS, catheter was removed, tourniquet removed and Bacitracin applied. Animals received subcutaneous buprenorphine (1 mg/kg) for postoperative pain control. Animals were followed daily for distress caused by pain or retention (posturing, dietary habits, wound infection, abdominal distension, dry bedding indicating lack of voiding). The procedure from incision to closure of circumcision site took approximately 25 minutes per rat. Subjects were euthanized at pre-determined time points on postoperative days 2, 5 and 28 using CO_2_ overdose followed by cervical dislocation. In total, 21 rats were used for this study (n = 9 for unseeded group; n = 12 for MSC/CD34+ HSPC seeded group). Animal studies were approved by the Institutional Animal Care and Use Committee at the Stanley Manne Research Center, Chicago, with all methods performed in accordance with the relevant guidelines and regulations.

Urethral patency was assessed using four parameters: 1) daily assessment for clinical voiding patterns (i.e. no abdominal distension, wet bedding); 2) void spot on filter paper at the time of euthanization; 3) urethral catheterization using a 22Fr catheter after euthanization; and 4) abdominal dissection and confirmation of bladder decompression post-mortem.

### Tissue Preparation

Formalin-fixed tissue was gradually dehydrated and embedded in paraffin before generating 5 μm sections using a microtome as previously described^11^. Hematoxylin-eosin, Masson’s Trichrome and Picrosirius staining (Sigma-Aldrich, MO, USA) were performed using standard protocols as previously described^11^,^12^.

### Immunohistochemistry

Preparation of slides for immunofluorescence staining was performed as previously described^11^, and briefly consisted of slide incubation at 62**°**C for 30 minutes before boiling in antigen retrieval solution (0.01 M citrate, pH 6.0, 0.05% Tween-20) for 15 minutes, cooling to room temperature for 50 minutes, followed by blocking with bovine serum albumin (BSA) (Sigma-Aldrich; 5 mg/mL) for 15 minutes. Primary antibodies for myeloperoxidase (MPO) at 2 μg/mL (rabbit anti-rat), CD68 at 10 μg/mL (mouse anti-rat), TNFα at 9 μg/mL (rabbit anti-rat), γ-tubulin at 20 μg/mL (mouse anti-human), IL-1β at 20 μg/mL (rabbit anti-rat) and IL-10 at 5 μg/mL (rabbit anti-rat) in 1% BSA were incubated for 1 hour. After washing with phosphate-buffered saline (PBS), slides were incubated with secondary antibodies (Invitrogen Corp., CA, USA) for 30 minutes at a dilution of 1:400. Following this, slides were rinsed with PBS and mounted with Vectashield (Vector Laboratories, CA, USA). All primary antibodies were obtained from Abcam (MA, USA).

### Quantification of Substitution Urethroplasty Tissues

Brightfield and polarized microscopy images were analyzed using Adobe Photoshop CC (Adobe Systems Inc., CA, USA). Vascular quantification was achieved by analyzing the Masson’s Trichrome images, counting number of vessels, pixel area and diameter of vessels per high power field (HPF) on circumferential images (ten slides per sample, at 40× magnification periuthreally). Picrosirius slides were evaluated under polarized light, with images divided into red, green and blue channels, analyzing the area of red-yellow (collagen I) or green (collagen III) birefringence per HPF on circumferential images (ten slides per sample, at 40× magnification periuthreally).

For neutrophil, macrophage, and cytokine detection, image analysis was performed using ImageJ, version 1.46r (NIH, http://rsbweb.nih.gov/ij/), once again on circumferential images (ten slides per sample, at 40× magnification periuthreally). The integrated cell counting function was used after setting appropriate color threshold, determining percent positivity for immunofluorescence.

### Statistical Analysis

Means and standard error are presented, and significances calculated with Student’s *t* test or ANOVA. P values < 0.05 were considered statistically significant. Analysis was performed using SPSS 21 for MAC (IBM Corp., NY, USA).

## Results

### Surgical outcomes

A total of 21 rats underwent substitution urethroplasty (n = 9 in unseeded group; n = 12 in MSC/CD34+ HSPC group) with all subjects surviving to their pre-determined endpoint. All subjects but one (20/21, 95.2%) met both functional and anatomical measures for urethral patency at their endpoint. One subject in the seeded group was found to be in urinary retention at the 28-day endpoint, and had undergone urethroplasty by a different surgeon from other subjects, less familiar with the procedure. This rat was excluded from analysis given both surgical and functional differences from all other subjects.

### Stem Cell Proliferation

**Figure 2:**
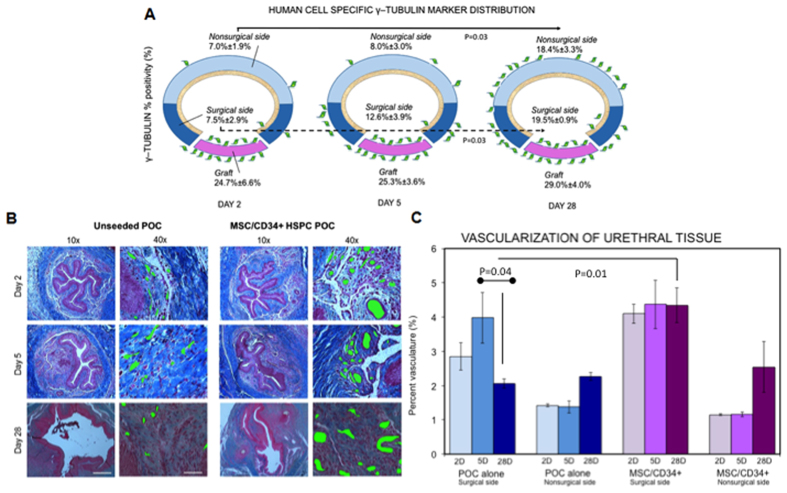
Stem cell survival, proliferation and neovascularization in urethral tissue. (**A**) Stem cells of human origin were identified immunohistochemically with γ-tubulin (represented by green cells on urethral model). γ-tubulin positivity remained stable in the region of the graft (pink), suggesting persistence of cells on the graft. Increasing levels of stem cells periurethrally (surgical side of urethra in dark blue, nonsurgical side of urethra in light blue) over time indicates both migration and proliferation of cells with time (p = 0.03 on surgical and nonsurgical sides of the urethra). (**B**) Masson’s Trichrome-stained images demonstrate differences in vessel number and size (vessels marked with green) over time between seeded and control animals at the surgical site. Vessels tended to increase in size in seeded animals with time, compared to persistently small vessels in the control group. In columns with 10× magnification, scale bar 200 μm. In columns with 40× magnification, scale bar 50 μm. (**C**) Increased vascularity in seeded groups compared to controls is demonstrated in percent vasculature (%V). The disparity between groups is statistically significant at 4 weeks (4.4% ± 0.5% seeded vs. 2.1% ± 0.1% control, p = 0.01).

Human bone marrow-derived MSC and CD34+ HSPCs in the area of substitution urethroplasty were identified using human-specific γ-tubulin antibody immunohistochemistry postoperatively as previously described^11^. Figure 2A depicts the proportion and distribution of γ-tubulin positive cells over time, showing the highest concentration of stem cells along the surface of the graft initially, with stability of γ-tubulin positivity over time (24.7% ± 6.6% on day 2, 25.3% ± 3.6% on day 5 and 29.0% ± 4.0% on day 28, p = 0.81). An increasing proportion of human γ-tubulin + cells was observed at the surgical site of urethroplasty over time (7.5% ± 2.9% on day 2, 12.6% ± 3.9% on day 5 and 19.5% ± 0.9% on day 28, p = 0.03) suggesting migration and proliferation of stem cells to the surgical site with healing. This effect is similarly seen periurethrally, on the contralateral (nonsurgical) side of the urethra. Unseeded groups were also analyzed as a control, demonstrating nominal background positivity (γ-tubulin positivity 2.0% on day 2, 0.33% on day 5 and 0.70% on day 28).

### Neovascularization

An increase in mean vascularity is seen in the immediate postoperative period (day 2 to 5) in all groups, although this initial change was not statistically significant (all p > 0.05). At these early time points, more robust neovascularization (assessed by vessels/mm^2^) was seen in the seeded (MSC/CD34+ HSPC) group as evidenced by disparity by day 5 postoperatively (199.5 ± 24.2 vessels/mm^2^ in seeded group vs. 90.7 ± 12.2 vessels/mm^2^ in unseeded group, p = 0.02). With longer follow-up, histologically, a change in size of vessels between groups is seen, with seeded animals demonstrating a shift towards fewer and larger-sized vessels compared to unseeded animals, in whom vessels remained relatively small (Fig. 2B). By later phases of wound healing at 4 weeks, percent vasculature is significantly higher at the surgical site in seeded animals compared to controls (Fig. 2C). When classifying vasculature by vessel size distribution, 69.0% ± 3.8% of vessels in the unseeded group were within the smallest size vessel grouping, while they made up 50.1% ± 4.7% of vessels seen in the seeded group.

### Modulation of Inflammation

**Figure 3:**
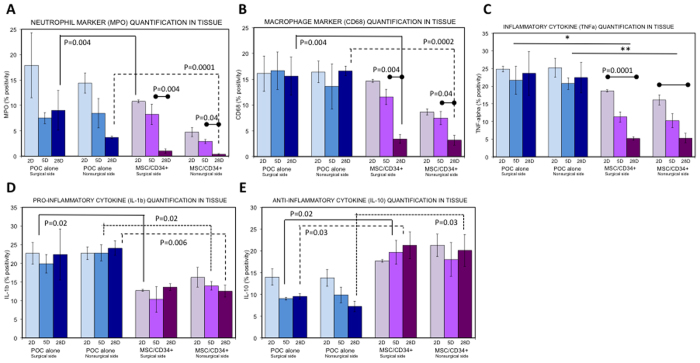
Cell-seeding with MSC/CD34 + HSPC demonstrates modulation of inflammation in the healing urethra. Myeloperoxidase (MPO) neutrophil marker, CD68 macrophage marker and inflammatory cytokines TNFα and IL-1β are decreased in seeded groups, while anti-inflammatory cytokine IL-10 is increased in seeded groups compared to controls. (**A**) By 4 weeks post-urethroplasty, seeded animals demonstrated a precipitous drop in MPO positivity on both surgical (8.2% ± 2.0% day 5 to 1.0% ± 0.4% day 28, p = 0.009) and nonsurgical (2.9% ± 0.4% day 5 to 0.4% ± 0.2% day 28, p = 0.001) sides of the urethra. MPO levels were significantly lower in the seeded groups. (**B**) While unseeded animals demonstrated a persistent elevation in CD68 positivity, seeded animals demonstrated significant reduction in CD68 positivity over time on both surgical (14.7% ± 1.3% day 2, 11.6% ± 1.5% day 5, 3.4% ± 0.9% day 28, p = 0.004) and nonsurgical (8.7% ± 0.6% day 2, 7.4% ± 1.3% day 5, 3.2% ± 1.0% day 28, p = 0.04) sides of the urethra. (**C**) TNFα cytokine levels showed a steady decline in seeded groups over time, while levels in control groups remained stably elevated. At 2, 5 and 28 days postoperatively, seeded animals had significantly lower levels of TNFα than controls on surgical (single asterisk: all p < 0.05) and nonsurgical (double asterisk: all p < 0.03) sides of the urethra. (**D**,**E**) IL-1β and IL-10 levels remained stable over time in both seeded and unseeded animals. Pro-inflammatory cytokine IL-1β is significantly lower in seeded urethras (**D**), while anti-inflammatory IL-10 is present at greater concentration in seeded animals than controls (**E**).

MSC/CD34+ HSPC seeding demonstrated potent anti-inflammatory effects throughout the healing process, as early as 2 and 5 days postoperatively, with persistent effects with extended follow-up, up to 28 days. This effect is seen across multiple markers with regards to both trend and absolute levels of pro-inflammatory cytokines TNFα (seeded: 5.1% ± 0.5% versus unseeded: 23.6% ± 6.2% on day 28, p = 0.017) and IL-1β (seeded: 13.6% ± 0.9% versus unseeded: 22.4% ± 6.8% on day 28, p = 0.19), neutrophil marker MPO (seeded: 1.0% ± 0.4% versus unseeded: 9.0% ± 3.9% on day 28, p = 0.06) and macrophage marker CD68 (seeded: 3.4% ± 0.9% versus unseeded: 15.6% ± 3.7% on day 28, p = 0.01) and the anti-inflammatory cytokine IL-10 (seeded: 21.3% ± 3.1% versus unseeded: 9.5% ± 0.7% on day 28, p = 0.03). All components of the inflammatory phase demonstrated a statistically significant decrease in the healing urethra in seeded animals over time, while anti-inflammatory and pro-regenerative cytokine IL-10 was upregulated. In comparison, while neutrophils showed a slight decrease by day 5 in the unseeded subjects, no further significant drop is seen and TNFα and macrophage levels remained stably elevated throughout the entire healing process in the unseeded group. Comparative trends in marker levels on are shown in Fig. 3.

### Tissue Maturation and Scar Formation

**Figure 4:**
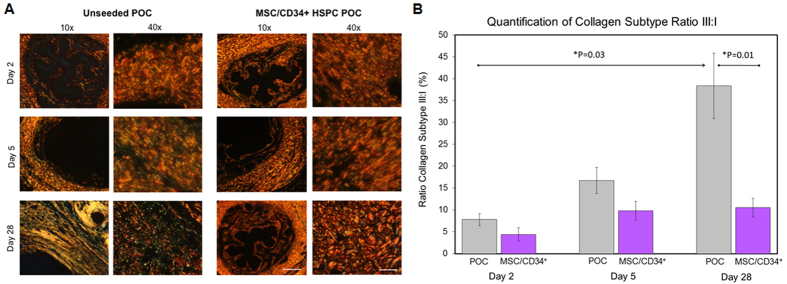
MSC/CD34+ HSPC results in earlier transition to wound remodeling and maturation-different proportion of collagen subtypes. (**A**) Polarized light photomicrographs of picrosirius staining demonstrates a relative increase in poorly organized, collagen type III (green birefringence) in unseeded urethroplasty over time, while seeded animals transition to an increase in collagen type I (red yellow birefringence) arranged in thick, organized bundles by 4 weeks. In columns with 10× magnification, scale bar 200 μm. In columns with 40× magnification, scale bar 50 μm. (**B**) Unseeded urethral wounds showed a continuous rise in ratio of collagen type III:I without reaching a plateau (7.8 ± 1.4 day 2, 16.7 ± 3.0 day 5, 38.4 ± 7.4 day 28, p = 0.009). In contrast, the cell-seeded urethra collagen ratio remained relatively stable (4.4% ± 1.5 day 2, 9.8 ± 2.1% day 5, 10.5 ± 2.1 day 28, p = 0.1).

Tissue maturation was determined by the ratio of collagen III to collagen I in the periurethral region using picrosirius staining. We demonstrate a significantly elevated and increasing ratio of collagen III:I during urethral healing in the unseeded group in contrast to the stabilized and lower ratio seen in the seeded animals (Fig. 4). Early in the postoperative period, the ratio of collagen III:I is similar in both groups (p values > 0.05). In the seeded group, there is a slight, nonsignificant rise in ratio from day 2 to 5 (4.4 ± 1.5 on day 2; 9.8 ± 2.1 on day 5, p = 0.15), which subsequently remains unchanged (10.5 ± 2.1 on day 28). In contrast, the unseeded group demonstrated a continuous rise in ratio of collagen III:I throughout healing (7.8 ± 1.4 on day 2, 16.7 ± 3.0 on day 5 and 38.4 ± 7.4 on day 28, p = 0.03), with a ratio significantly higher in the unseeded than seeded group at 28 days postoperatively (p = 0.012).

## Conclusions

The balance between essential versus prolonged inflammation seen with post-surgical healing is required for an environment promoting proper tissue regeneration and limiting sequelae of pathologic inflammation and scar formation. While the tissue injury with surgery stimulates an innate immune response including the recruitment of granulocytes and macrophages, abnormally persistent elevation of a pro-inflammatory response in healing tissues may result in poor tissue reformation and immature architectural remodeling^13^,^14^.

Despite advances in tissue engineering over the last several decades, use of graft tissues remain limited to date by immunogenic host response, as well as limitations of vascularity and availability and variability of scaffolding tissues, all factors which increase graft failure. Although MSC are known to exhibit potent anti-inflammatory properties and CD34+ HSPCs have demonstrated the promotion of angiogenesis in ischemic myocardium, it is not until recently that the combination of these stem cell populations has been investigated^9^,^10^,^15^,^16^,^17^. Several recent studies have highlighted the co-transplantation of MSC and/or CD34+ HSPC with POC scaffolds in the rat bladder augmentation model demonstrating a synergistic effect on modulation of inflammation, promotion of neovascularization and urothelial growth^9^,^10^,^11^. These effects in the healing bladder make the application of this cell/scaffold composite highly attractive in the healing urethra.

To our knowledge, this work demonstrates the establishment of a novel substitution urethroplasty model in rats corroborated by data that supports both the feasibility of the surgical procedure and urethral patency at a minimum of four weeks. Urethral wound healing has been previously characterized in rats, and provides a framework for comparison with the addition of POC scaffold and cell-seeding^18^. Furthermore, we report the first application of cell-seeding the synthetic scaffold POC with MSC and CD34+ HSPC in the urethra, with results suggesting robust modulation of inflammation, favorable collagen remodeling and possible accelerated vascular maturation.

The anti-inflammatory effect of MSC/CD34+ HSPC cells was seen consistently across multiple inflammatory markers at both early and late urethral healing (Fig. 3). Specifically, cell-seeded urethras demonstrated a 1.3 and 1.7-fold reduction in inflammatory cytokine TNFα levels and neutrophil migration compared to unseeded animals as early as 2 days postoperatively, respectively. This early divergence in inflammatory response between seeded and unseeded subjects became increasingly evident over time, ultimately resulting in a 4.6 and 8.8-fold reduction in TNFα and neutrophil levels at 4 weeks. This modulatory effect was mirrored in the 1.4-fold reduction in macrophage levels at 5 days, progressing to a 4.6-fold differential by 4 weeks. These results are further supported by a downregulation of pro-inflammatory cytokine IL-1β and concomitant upregulation of anti-inflammatory and pro-regenerative cytokine IL-10. The anti-inflammatory effects of MSC/CD34+ HSPC were exhibited first in the immediate operative field, or surgical side of urethra, but then also became evident throughout the periurethral tissue, with delayed effects echoed on the nonsurgical side of the urethra.

Protracted or inappropriate inflammation in the setting of healing tissues can result in pathologic wound healing and scar formation, best illustrated in lichen sclerosis, which is a condition characterized by chronic inflammation resulting in progressive and recurrent urethral strictures^19^,^20^. Much of the literature regarding histologic characterization of hypertrophic scars exists in the context of hypertrophic burn scars and keloids, with studies suggesting increased collagen III relative to I in hypertrophic or pathologic scar formation compared to normal skin and normal, mature scars^21^,^22^,^23^,^24^. While collagen III production by myofibroblasts is seen in the proliferative phase of wound healing, a shift towards rearrangement in an organized fashion with collagen I is an indicator of wound maturation and remodeling with increase in tensile strength^25^. MSC/CD34+ HSPC-seeded urethras demonstrated a physiologic initial increase in collagen III:I content in the early stages of urethral healing, similar to controls. However, a striking difference evolved by 4 weeks into the urethral healing process, with a 3.6-fold reduction in collagen III:I ratio in seeded animals compared to controls, suggesting that while substitution urethroplasty may induce prolonged immature scar formation (indicated by persistent production of collagen III), cell-seeding limits scar formation and instead enhances transition to mature collagen architecture (Fig. 4).

Sufficient vascularization of grafted tissue is imperative for adequate healing and exchange of nutrients and gases, preventing graft necrosis^10^,^26^. An adequate graft material would therefore allow for vascularization permitting graft and cell survival, while an ideal graft may enhance neovascularization. In all groups, an initial increase in vascularity is seen on days 2 and 5, as wound be expected in the acute postsurgical field. With further wound healing, MSC/CD34+ HSPC seeded animals developed more robust vasculature by 4 weeks postoperatively. Histologically, a change in vascular profile is evident in which initially small, numerous vessels progress to larger, more mature vessels later in the healing process in seeded groups. In contrast, this progression is not demonstrated in the control group.

This study provides an unprecedented examination of the histological changes after substitution urethroplasty in a rat model, characterizing the striking and beneficial effects on urethral healing in the presence of MSC/CD34+ HSPC. Only specific aspects of the innate immune system were studied, providing important, albeit limited, information; therefore, other parts of the immune response must be examined to fully characterize the relationship between cell-seeding and tissue regeneration. While examination of collagen ratio as a proxy for maturation and remodeling of scar may limit this study, long-term outcomes assessing clinical obstruction will be a more telling outcome in future studies. Furthermore, while this study provides conceptual groundwork for strategies to optimize tissue engineering in urethroplasty, as with any animal model, translation of these results using a rat model will require further animal and eventual human studies.

Cell-seeding of the POC scaffold with the combination of MSC and CD34+ HSPC demonstrates a striking modulation of the inflammatory milieu, increased vascularization and acceleration of scar maturation and remodeling. These effects are demonstrable as early as 2 and 5 days postoperatively compared to controls, with continued favorable effects with increasing divergence from controls at 4 weeks. Favorably altering the urethral process to prevent pathologic inflammation and hypertrophic scar, while enhancing blood supply to graft tissue could have wide-reaching implications for the future of substitution urethroplasty and reduction of stricture recurrence. We furthermore demonstrate both feasibility and efficacy of substitution urethroplasty in a novel rat model.

## Additional Information

**How to cite this article**: Liu, J. S. *et al*. Bone Marrow Stem/Progenitor Cells Attenuate the Inflammatory Milieu Following Substitution Urethroplasty. *Sci. Rep.* **6**, 35638; doi: 10.1038/srep35638 (2016).
